# Evolution of Br⋯Br contacts in enantioselective molecular recognition during chiral 2D crystallization

**DOI:** 10.1038/s41467-022-33446-y

**Published:** 2022-10-04

**Authors:** Zhen-Yu Yi, Xue-Qing Yang, Jun-Jie Duan, Xiong Zhou, Ting Chen, Dong Wang, Li-Jun Wan

**Affiliations:** 1 CAS Key Laboratory of Molecular Nanostructure and Nanotechnology, CAS Research/Education Center for Excellence in Molecular Sciences, Beijing National Laboratory for Molecular Sciences (BNLMS), Institute of Chemistry, Chinese Academy of Sciences, Beijing, 100190 China; 2 University of Chinese Academy of Sciences, Beijing, 100049 China; 3 College of Chemistry and Chemical Engineering, Hubei University, Wuhan, 430062 China; 4 Beijing National Laboratory for Molecular Sciences (BNLMS), College of Chemistry and Molecular Engineering, Peking University, Beijing, 100871 China

**Keywords:** Surface assembly, Halogen bonding

## Abstract

Halogen-mediated interactions play an important role in molecular recognition and crystallization in many chemical and biological systems, whereas their effect on homochiral versus heterochiral recognition and crystallization has rarely been explored. Here we demonstrate the evolution of Br⋯Br contacts in chiral recognition during 2D crystallization. On Ag(100), type I contacts prevail at low coverage and lead to homochiral recognition and the formation of 2D conglomerates; whereas type II contacts mediating heterochiral recognition are suppressed at medium coverage and appear in the racemates induced by structural transitions at high coverage. On Ag(111), type I contacts dominate the 2D crystallization and generate 2D conglomerates exclusively. DFT calculations suggest that the energy difference between type I and type II contacts is reversed upon adsorption due to the substrate induced mismatch energy penalty. This result provides fundamental understanding of halogen-mediated interactions in molecular recognition and crystallization on surface.

## Abstract (web summary)

Halogen-mediated interactions control molecular recognition in many chemical and biological systems. Here, the authors demonstrate two types of Br⋯Br contacts and their importance in chiral on-surface crystallization.

## Introduction

Since the separation of sodium ammonium tartrate by Pasteur in 1847, chiral crystallization has attracted extensive attention due to its importance in nature and in many fields of science. Of particular interest in recent years has been the 2D crystallization of chiral molecules on solid surfaces^1–5^. It is not only of significance for developing chiral surfaces but also contributes to a mechanistic understanding of the interaction and recognition of chiral molecules. In general, chiral molecules can recognize either homochirally or heterochirally during crystallization on the surface, resulting in three crystallization outcomes, i.e., 2D conglomerates, 2D racemates, and 2D random solutions^6^. Until now, however, it is a great challenge to predict whether homochiral or heterochiral recognition is preferred, and what kind of outcomes are generated in 2D crystallization. It is of fundamental importance to understand the relationship of noncovalent interactions with homochiral versus heterochiral recognition and crystallization.

Halogen-mediated weak interactions are widely present in the molecular recognition and crystallization processes in many chemical and biological systems^7–10^. Recently, the development of high-resolution microscopes, including scanning tunneling microscopy (STM), makes it possible to study the halogen-mediated interactions in molecular recognition and assembly on surfaces at the sub-molecular level^11^, for example, identifying the halogen-mediated interactions and their basic characteristics^12–14^, understanding the dependence of halogen bond on molecular structure and substrate^15,16^, and assessing the contribution of halogen bond to molecular assemblies^17,18^. Furthermore, a number of supramolecular architectures like open porous networks^19,20^, ordered fractal patterns^21^, and highly complex tessellations^22^, have been fabricated on surfaces based on halogen-mediated interactions. Nevertheless, the effect of halogen-mediated interactions on the homochiral versus heterochiral recognition and crystallization on the surface has seldom been investigated.

**Fig. 1:**
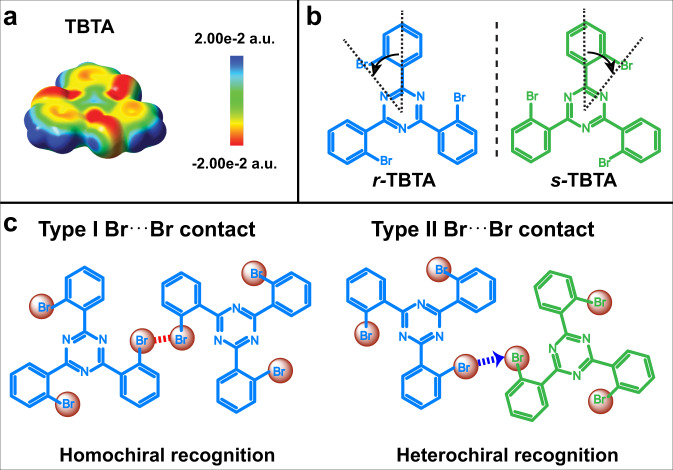
Electrostatic potential of TBTA and its adsorption configuration and intermolecular interactions on the surface. **a** Calculated electrostatic potential map of TBTA. **b** Molecular structures of *r*-TBTA and *s*-TBTA. For clarity, *r*-TBTA and *s*-TBTA are colored blue and green, respectively. **c** Homochiral and heterochiral recognition of TBTA through type I and type II Br⋯Br contacts, respectively. The red dots represent the Br atoms. The type I and type II Br⋯Br contacts are denoted with a red dashed line and blue dashed arrows, respectively.

Herein, we demonstrate the effect of Br⋯Br contacts on the enantioselective molecular recognition and 2D crystallization of prochiral molecule 2,4,6-tris(2-bromophenyl)-1,3,5-triazine (TBTA) on surfaces. Figure 1a is the electrostatic potential map of TBTA, where characteristic positive *σ*-hole and negatively charged equatorial zone are revealed on bromine atoms. The prominent feature of TBTA is that the bromine atoms are at the *ortho*-positions rather than the *meta*- or *para*- positions as is common in literatures^23–27^. To minimize the steric hindrance between the bromine atoms, TBTA adopts a *C*_3h_ conformation (Supplementary Fig. 1) and thus has two adsorbed conformers with opposite chirality on surfaces, named *r*-TBTA and *s*-TBTA, respectively (Fig. 1b). In addition, the bromine atoms block the central triazine ring so that it cannot participate in intermolecular interactions. They also prevent the formation of multiple Br⋯Br synthons^11^, for example, the Br-3 synthons^14,28^, due to intermolecular steric hindrance. In this way, the interactions between TBTA molecules are constrained to type I and type II Br⋯Br contacts, corresponding to the homochiral and heterochiral intermolecular recognition respectively (Fig. 1c). We investigated the recognition and assembly of TBTA on Ag(100) and Ag(111) at different coverages. STM imaging demonstrated that TBTA molecules interact via type I or type II Br⋯Br contacts, giving rise to homochiral or heterochiral dimeric motifs, respectively. Moreover, we revealed a relationship between the presence of type I or/and type II Br⋯Br contacts and the crystallization outcomes. The preferential occurrence of type I or type II Br⋯Br contacts leads to the formation of 2D homochiral honeycomb networks (HCBs) or 2D racemic zigzag row structures (ZZRs); and the co-occurrence of both types generates 1D-disordered racemic structures on the surface. DFT calculations were performed to understand the evolution of type I or/and type II Br⋯Br contacts and their correlation to the chiral recognition and crystallization of TBTA on the surface.

## Results

### The adsorption of TBTA on Ag(100) at 0.05 ML

We firstly explored the deposition of TBTA at very low coverage to understand the adsorption of TBTA on Ag(100). In the experiments, TBTA molecules were deposited onto a cold Ag(100) surface (150–200 K), followed by annealing at room temperature for 15 min. After that, the TBTA/Ag(100) sample was transferred to the STM stage hold at 4.3 K for STM measurements.

**Fig. 2:**
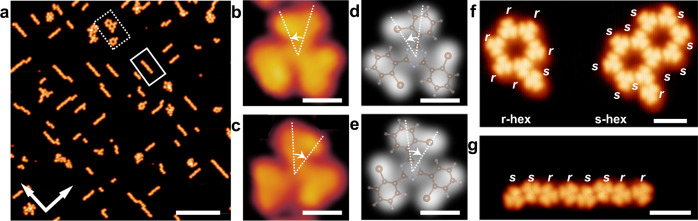
Adsorption of TBTA on Ag(100). **a** STM image of TBTA/Ag(100) with coverage of 0.05 ML. **b**, **c** High-resolution STM images of an individual *r*-TBTA and *s*-TBTA on Ag(100). **d**, **e** Simulated STM images of *r*-TBTA and *s*-TBTA on Ag(100). The molecular structures of *r*-TBTA and *s*-TBTA are superimposed on the images for comparison. **f**, **g** Amplified STM images of the molecular rings and the chains in panel (**a**). Tunneling conditions: **a** *V*_bias_ = 1.0 V, *I*_t_ = 50 pA. **b**, **c** *V*_bias_ = −0.4 V, *I*_t_ = 100 pA. **f**, **g** *V*_bias_ = 1.0 V, *I*_t_ = 50 pA. Scale bars: **a** 20 nm. **b**–**e** 0.5 nm. **f**, **g** 2 nm.

Figure 2a is an STM image of the TBTA/Ag(100) with a coverage of 0.05 ML. Some regular molecular rings and chains can be recognized, as depicted with the dashed and the solid rectangles, respectively. The lines connecting the centers of two adjacent pores in the molecular rings cross ±7° with the <$0\bar{1}1$> direction of Ag(100). And the molecular chains are along the <$0\bar{1}1$> direction of Ag(100). It is implied that the substrate lattice plays an important role in the adsorption and intermolecular recognition of TBTA on Ag(100).

*r*-TBTA and *s*-TBTA can be clearly resolved in the STM images, as shown in Fig.2b, c. The molecules appear as three-leaf windmills, in which each leaf corresponds to a bromophenyl moiety. Their experimental STM images are in good agreement with the simulated STM images (Fig. 2d, e). Figure 2f, g are amplified STM images of the molecular rings and chains. It is revealed that the molecular rings are homochiral and contain either only *s*-TBTA or *r*-TBTA; whereas the molecular chains contain both *r*-TBTA and *s*-TBTA.

It has been reported that the dehalogenation reaction of phenyl bromide groups occurs already at room temperature or slightly above room temperature on silver surfaces and gives rise to organometallic intermediates^29–32^. In the present study, we found that TBTA molecules remain intact after room temperature annealing. Firstly, we conducted stepwise annealing of TBTA/Ag(100) and TBTA/Ag(111) and found that 1D chains of organometallic intermediates appear on the surface after annealing at 370 K (Supplementary Fig. 2), accompanied by the observation of Br atoms on the surface. Furthermore, we employed XPS to elucidate the chemical states of TBTA on Ag(100) at different temperatures. The results confirm that dehalogenation and the formation of organometallic intermediates do not occur up to room temperature on Ag(100) (Supplementary Fig. 3).

### The 2D homochiral HCBs of TBTA on Ag(100)

Then, we gradually increased the coverage of TBTA to explore the 2D crystallization of TBTA on Ag(100). It was found that the molecular rings propagate on the surface and finally form 2D extended homochiral HCBs. However, the molecular chains do not grow with the coverage.

**Fig. 3:**
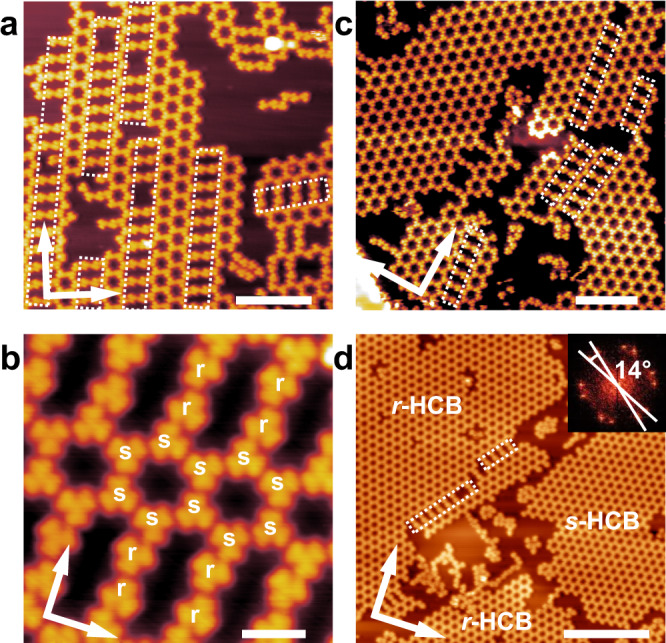
Formation of the 2D homochiral HCBs of TBTA on Ag(100). **a, b** STM images of TBTA/Ag(100) with coverage of 0.4 ML. **c**, **d** STM images TBTA/Ag(100) with coverage of 0.8 and 0.9 ML, respectively. Tunneling conditions: **a**, **b** *V*_bias_ = 0.5 V, *I*_t_ = 90 pA. **c** *V*_bias_ = 0.7 V, *I*_t_ = 100 pA. **d** *V*_bias_ = −0.5 V, *I*_t_ = 100 pA. Scale bars: **a** 10 nm. **b** 2 nm. **c** 10 nm. **d** 20 nm.

At 0.4 ML, porous islands consisting of molecular rings, as well as a small fraction of the molecular chains, can be observed simultaneously (large scale STM image is shown in Supplementary Fig. 4). Figure 3a is an amplified STM image of the porous islands, in which porous stripes and small patches of HCBs are revealed. The formation of the porous stripes indicates the propagation of the molecular rings is anisotropic. Moreover, the orientation of the molecular rings relative to substrate lattice is kept during growth that both the strips and the unit cell of the HCBs exhibit an offset of ±7° with respect to the <$0\bar{1}1$> direction of the substrate. Some molecular pairs are embedded between adjacent porous strips or HCBs (marked with white dashed lines). High-resolution STM images indicate that the porous strips and the HCBs are homochiral (Fig. 3b). The embedded molecular pairs also are homochiral; however, they exhibit opposite handedness to the porous strips or HCBs they attached.

Further increasing the molecular coverage leads to a gradual growth of the porous strips and the small patches of HCBs to 2D extended HCBs on the surface. Figures 3c, d are STM images obtained at 0.8 and 0.9 ML, respectively. Nearly a full monolayer of the 2D extended HCBs is revealed. In the monolayer, two enantiomorphic domains of HCBs were distinguished, named *r*-HCBs and *s*-HCBs, respectively. The unit cells of *r*-HCBs and *s*-HCBs have an offset of +7° and −7° from the <$0\bar{1}1$> direction of Ag(100), respectively (Inset is the 2D-FFT image).

**Fig. 4:**
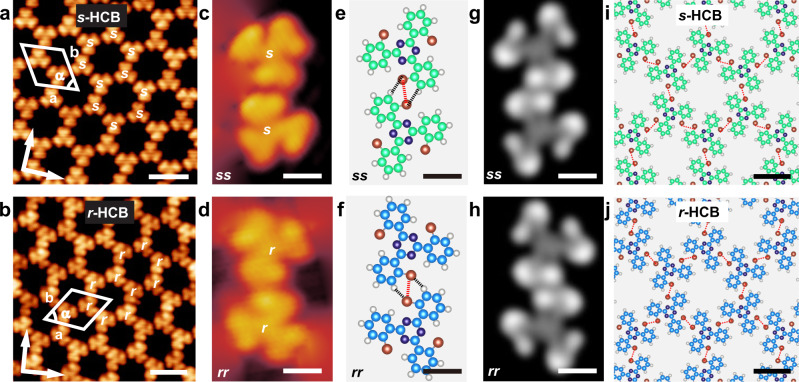
STM images and molecular models of the 2D homochiral HCBs. **a**, **b** High-resolution STM images of the *s*-HCBs and the *r*-HCBs, respectively. **c**, **d** Amplified STM images of the homochiral *ss*- and *rr*- dimers. **e**, **f** DFT optimized molecular models of the homochiral *ss*- and *rr*- dimers. **g**, **h** Simulated STM images of the homochiral *ss*- and *rr*- dimers. **i**, **j** Optimized molecular models of the *s*-HCBs and *r*-HCBs. Tunneling conditions: **a** *V*_bias_ = −0.4 V, *I*_t_ = 100 pA. **b** *V*_bias_ = 0.1 V, *I*_t_ = 60 pA. **c** *V*_bias_ = −0.4 V, *I*_t_ = 60 pA. **d** *V*_bias_ = −0.03 V, *I*_t_ = 100 pA. Scale bars: **a**, **b** 2 nm. **c**–**h** 0.5 nm. **i**, **j** 1 nm.

Figure 4a, b are high-resolution STM images of the *s*-HCBs and *r*-HCBs, respectively. A unit cell is superimposed in the images, in which a = b = 2.1 ± 0.1 nm and α = 60 ± 1°. The molecular density of the 2D homochiral HCBs is calculated to be 0.52 molecule/nm^2^. The elementally structural units of the *s*-HCBs and *r*-HCBs are homochiral *ss*- and *rr*-dimers, respectively (Fig. 4c, d). To understand the interactions within the homochiral *ss*- and *rr*-dimers, we performed DFT calculations. As shown in the optimized molecular models (Fig. 4e, f), the molecules are antiparallel to each other and interact via the bromophenyl moieties. The C-Br⋯Br angles are identical, which are 95°. The Br⋯Br bond is 3.70 Å in length. These results suggest the molecules interact mainly through type I Br⋯Br contacts, complemented by hydrogen bonding between the Br and *α*-H atoms (black dashed lines). Simulated STM images of *ss*- and *rr*- dimers agree well with their experimental STM images (Fig. 4g, h). Figure 4i, j are the optimized molecular models of the *s*-HCBs and the *r*-HCBs, respectively. It is indicated that type I Br⋯Br contacts dominate the intermolecular recognition in the 2D homochiral HCBs, while the type II Br⋯Br contacts are totally suppressed. The optimized molecular models match well with the experimental STM images (Supplementary Fig. 5). The simulated STM images of the *s*-HCBs and the *r*-HCBs based on the optimized molecular models are shown in Supplementary Fig. 6.

### Structural transition from the 2D homochiral HCBs to the 2D racemic ZZRs on Ag(100)

**Fig. 5:**
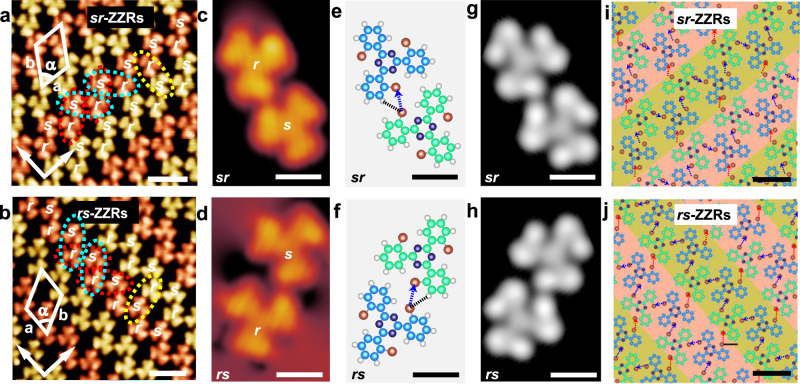
STM images and molecular models of the 2D racemic ZZRs. **a**, **b** High-resolution STM images of the *sr*-ZZRs and *rs*-ZZRs, respectively. For clarity, the adjacent zigzag rows are painted in different colors. **c**, **d** Amplified STM images of the heterochiral *sr*-dimer and the *rs*-dimer. **e**, **f** DFT optimized molecular models of the heterochiral *sr*-dimer and the *rs*-dimer. **g**, **h** Simulated STM images of the heterochiral *sr*-dimer and the *rs*-dimer. **i**, **j** Optimized molecular models of the *sr*-ZZRs and *rs*-ZZRs. The blue arrows and the red arrows denote the Br⋯Br contacts within and between the rows, respectively. Tunneling conditions: **a** *V*_bias_ = −0.3 V, *I*_t_ = 50 pA. **b** *V*_bias_ = 0.3 V, *I*_t_ = 50 pA. **c** *V*_bias_ = −0.4 V, *I*_t_ = 50 pA. **d** *V*_bias_ = −0.4 V, *I*_t_ = 100 pA. Scale bars: **a**, **b** 2 nm. **c**–**h** 0.5 nm. **i**, **j** 1 nm.

After obtaining nearly a monolayer of the 2D homochiral HCBs, further increase of the molecular coverage induces a structural transition to the 2D racemic ZZRs (Supplementary Fig. 7). Figure 5a, b are high-resolution STM images of the 2D racemic ZZRs. Zigzag rows can be clearly distinguished, which are oriented at ±2° with respect to the <$0\bar{1}1$> direction of Ag(100). Within the zigzag rows, the *s*-TBTA and *r*-TBTA pack alternately and form two mirror-imaged heterochiral *sr*- and *rs*-dimers, denoted with the blue dashed ellipses and the red dashed ellipses, respectively. Figure 5c, d are the magnified STM images of the *sr*- and *rs*- dimers. Although they are racemic in composition, they are enantiomorphic to each other in view of the relative alignment of the molecules. This result is reasonable because the heterochiral dimers are asymmetric in geometry. Therefore, two mirror-imaged molecular motifs can be generated when they are confined on the surface^33^. These racemic zigzag rows can only be observed at coverages around a monolayer. They are a result of the coverage-induced structural transition rather than the enlargement of the initially formed molecular chains.

Analogously, although the 2D ZZRs are racemic in composition, they have two enantiomorphous domains considering the relative alignment of the neighboring zigzag rows. Similar racemic zigzag rows of heptahelicene have been observed on Cu(111) surface^2^. For clarity, the adjacent zigzag rows are painted in different colors. The yellow dashed ellipse marks a pair of interacting molecules in the adjacent rows. In the *sr*-ZZRs (Fig. 5a) and *rs*-ZZRs (Fig. 5b), the interacting molecules form *sr*-dimer and *rs*-dimer, respectively. Therefore, *sr*-ZZRs and *rs*-ZZRs are enantiomorphic to each other. A unit cell was superimposed onto the STM images, in which a = 1.8 ± 0.1 nm, b = 2.2 ± 0.1 nm, and α = 62 ± 2°. The molecular density of the racemic zigzag row structures is calculated to be 0.57 molecule/nm^2^, which is higher than that of the 2D homochiral HCBs.

Figure 5e–h are the optimized molecular models and the corresponding simulated STM images of the heterochiral *sr*- and *rs*- dimers. It is revealed that the interacted C-Br bonds are almost perpendicular to each other. The length of the Br⋯Br bond is 3.68 Å. And the C-Br⋯Br angles are 110° and 147°, respectively. The results suggest the formation of the heterochiral dimers is mainly directed by the type II Br⋯Br contacts, the real Br⋯Br halogen bonds according to the definition of IUPAC^10^. The C-Br⋯Br angles slightly deviate from the ideal angles, which may be due to a snapping of the molecules to the substrate lattice and the steric hindrance effect between the molecules^16^. In addition, the Br⋯H hydrogen bonds play a synergistic role in the heterochiral recognition of TBTA molecules as well. Figure 5i, j are the optimized molecular models of the 2D racemic *sr*-ZZRs and *rs*-ZZRs. It can be seen that the ZZRs are mediated by type II Br⋯Br contacts and synergetic Br⋯H interactions, while the type I Br⋯Br contact is disappeared. This result is completely different from the 2D homochiral HCBs, in which type I Br⋯Br contacts dominate the intermolecular interactions and recognition. Supplementary Fig. 8 reveals good agreement between the optimized molecular models and the experimental STM images of the heterochiral dimers and the *sr*-ZZR. We also simulated the STM images of the 2D racemic ZZRs, which exhibit good agreement with the experimental results as well (Supplementary Fig. 9).

### Structural transition from the 2D racemic ZZRs to the 1D-disordered racemic structures on Ag(100)

Interestingly, 1D-disordered racemates, a distinct racemic phase rarely observed^34,35^, emerge on the surface when the coverage of TBTA is above a monolayer (large scale STM image is shown in Supplementary Fig. 10).

**Fig. 6:**
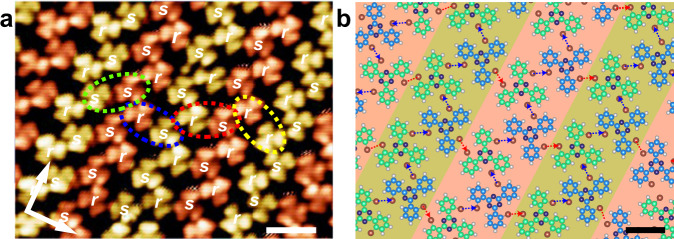
STM image and molecular model of the 1D-disordered racemates. **a** STM image of the 1D-disordered racemic structures was obtained at 1.1 ML. For clarity, the adjacent zigzag rows are painted in different colors. **b** Molecular model of the 1D-disordered racemic structures. Tunneling conditions: *V*_bias_ = −0.5 V, *I*_t_ = 100 pA. Scale bars: **a** 2 nm. **b** 1 nm.

As shown in the high-resolution STM image (Fig. 6a), *s*-TBTA and *r*-TBTA alternately distribute within the zigzag rows in an ordered manner, which is exactly the same as that in the 2D racemic ZZRs. However, the chiral recognition mode between adjacent zigzag rows in the 1D-disordered racemic structures is quite diverse, which is in sharp contrast to the uniform heterochiral *sr*- and *rs*-recognition in the *sr*-ZZRs and *rs*-ZZRs. All of the four molecular dimers, i.e., the *ss*-, *sr*-, *rs*-, and *rr*- dimers can be formed by the interacting molecules in the neighboring row, as marked with the green, the blue, the red, and the yellow dashed ellipses, respectively. It is clear that although type II Br⋯Br halogen bonding governs the molecular interactions and recognition within the rows, both type I and type II contacts are available for the interacting molecules in the adjacent rows (Fig. 6b). Therefore, the alignment of the adjacent zigzag rows is flexible, which is suggested to responsible for the emerging of the 1D-disordered racemic structures. We counted the number of molecules in Fig. 6a and roughly estimated the molecular density of the 1D-disordered racemic structures. The result indicates that the molecular density of the 1D-disordered racemic structures shown in Fig. 6a is about 0.58 molecule/nm^2^, which is slightly higher than that of the 2D racemic ZZRs.

## Discussion

**Fig. 7:**
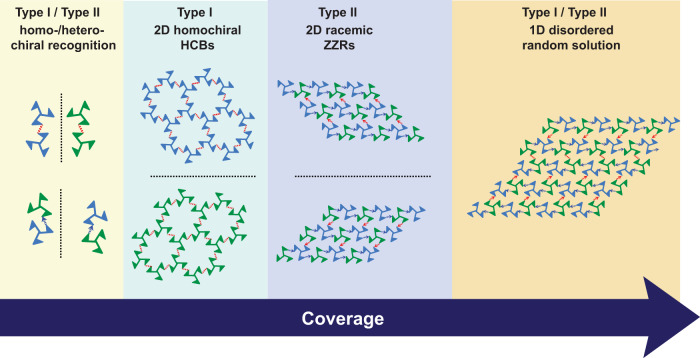
Evolution of Br⋯Br contacts during the 2D crystallization. The scheme shows the occurence of Br⋯Br contacts and the assembly available with the increase of the molecular coverage.

The 2D crystallization of TBTA on the Ag(100) surface is significantly influenced by coverage, as shown in Fig. 7. Type I and type II Br⋯Br contacts co-occur at very low coverage and mediate the homochiral and heterochiral recognition of TBTA, respectively. Molecular rings with only type I contacts and molecular chains with both type I and type II contacts coexist on the surface. At the medium-coverage stage, the homochiral molecular rings propagate on the surface via type I Br⋯Br contacts and finally form 2D homochiral HCBs. However, the molecular chains do not enlarge and are completely compressed eventually. After that, a phase transition occurs when the molecular coverage is higher than a monolayer. The 2D homochiral HCBs with type I contacts transform into 2D racemic ZZRs with type II contacts, and subsequently transform into 1D-disordered racemic structures containing both type I and type II contacts upon increasing the coverage.

In general, type I halogen⋯halogen contact is considered to be established through the dispersion-like attraction between the halogen atoms^36,37^. It is not a halogen bonding according to the IUPAC definition but arises from close-packing requirement^10^. In contrast, type II halogen⋯halogen contact is a real halogen-bonding between the *σ*-hole of one halogen atom and the negatively charged equatorial zone of a neighboring halogen atom^38,39^. Numerous studies demonstrated that type II contact plays a dominant role in molecular recognition and crystallization^11,14,27^. In the present study, the type I Br⋯Br contacts occur at very low coverage and are preferred over the type II Br⋯Br contacts at coverage below 0.9 ML. On the contrary, the type II Br⋯Br contacts are suppressed at coverage below 0.9 ML and occur in the 2D racemates produced by a structural transition in high coverage. This result is in sharp contrast to the general understanding of type I and type II halogen⋯halogen contacts.

In order to understand the abnormal occurrence of type I and type II Br⋯Br contacts in the nucleation and growth stages of TBTA on Ag(100), we performed DFT calculations. Fundamentally, the adsorption and assembly of molecules on a surface are determined by a combination of intermolecular interactions and molecule-substrate interactions. Therefore, we first calculated the energies of the homochiral dimers and the heterochiral dimers on Ag(100) to understand their stability on the surface; then, we removed the Ag(100) surface and calculated the energy of the homochiral dimers and the heterochiral dimers to assess the intermolecular interactions. Details of the theoretical calculations are shown in the Supplementary. In short, we first optimized the adsorption configuration of a single *s*-TBTA molecule (Supplementary Fig. 11); then, we proposed three homochiral dimers (*ss*-1, *ss*-2, *ss*-3) and three heterochiral dimers (*rs*-1, *rs*-2, and *rs*-3) considering the orientation of the dimers relative to the substrate lattice (Supplementary Fig. 12), in which an *s*-TBTA adopts the optimized adsorption configuration; finally, we calculated the energies of the dimers. The results are summarized in Supplementary Table 1.

It is indicated that the energies of the homochiral dimers are lower than the energies of the heterochiral dimers. The result consists well with the experimental result that the homochiral intermolecular recognition is favored over the heterochiral recognition at the low-coverage and medium-coverage and thus results in the propagation of the molecular rings on the surface. Among the three homochiral dimers, the energies of *ss*-1 and *ss*-2 are comparable but lower than that of *ss*-3, which explains well the anisotropic growth of the porous strips. As to the heterochiral dimers, though the energies of rs-1 and rs-2 are much higher than that of the homochiral dimers, the energy of *rs*-3 is comparable to that of *ss*-3. Therefore, some heterochiral dimers can be formed together with the homochiral dimers, just as revealed by STM images.

However, after the removal of Ag(100), the energy of the *ss*-1 dimer is slightly higher than that of the *rs*-3 dimer. The results suggest that the stability of the *ss*-1 dimer relative to the *rs*-3 dimer is strengthened after adsorption on Ag(100). This result may be due to the better match of the *ss*-1 dimer to the substrate lattice compared to the *rs*-3 dimer. As shown in Fig. 1c and Supplementary Fig. 12, the type I Br⋯Br contact is centrosymmetric. Both molecules in the homochiral dimer nearly maintain the optimized molecular orientation with respect to the substrate except for a slight rotation and displacement. However, the type II Br⋯Br contact exhibits a bent geometry and is asymmetrical. Two TBTA molecules in a heterochiral dimer cannot adsorb on the Ag(100) surface with the optimized molecular orientation at the same time. As a result, the stability of the homochiral dimer relative to the heterochiral dimers increases after adsorption on Ag(100).

Similarly, the preferential formation of the 2D homochiral HCBs in the medium-coverage stage may also be related to the fact that the 2D HCBs mediated by the type I Br⋯Br contacts are highly symmetrical, in which each TBTA molecule adsorbed on the Ag(100) surface with optimized molecular orientation. It is difficult to calculate the energies of the periodic 2D homochiral HCBs and 2D racemic ZZRs on Ag(100) surface because they are incommensurate with the Ag(100) surface. For simplicity, we calculated the energies of the homochiral and the heterochiral triplet clusters on Ag(100), i.e., the *sss*-trimer/Ag(100) and the *srs*-trimer/Ag(100), respectively (Supplementary Fig. 13). It is demonstrated that the energy difference between the *sss*-trimer/Ag(100) and the *srs*-trimer/Ag(100) is about 0.16 eV (lower for the homochiral triplet cluster), which is increased compared to that between the *ss*-1/Ag(100) and *rs*-3/Ag(100). The result reflects the influence of the matching degree of molecular orientation of the clusters to the substrate on their stability. Furthermore, it explains well why the homochiral clusters can propagate on the surface, whereas the racemic triplet clusters with only type II Br⋯Br contacts have not been observed before the structural transition.

The phase transition at high molecular coverage is mainly density-driven, which has been well discussed in many literatures^5,40–42^. In the present study, the molecular density increases gradually from the 2D homochiral HCBs to the 2D racemic ZZRs and to the 1D-disordered racemic structures, which is consistent with the characteristics of a density-driven phase transition.

**Fig. 8:**
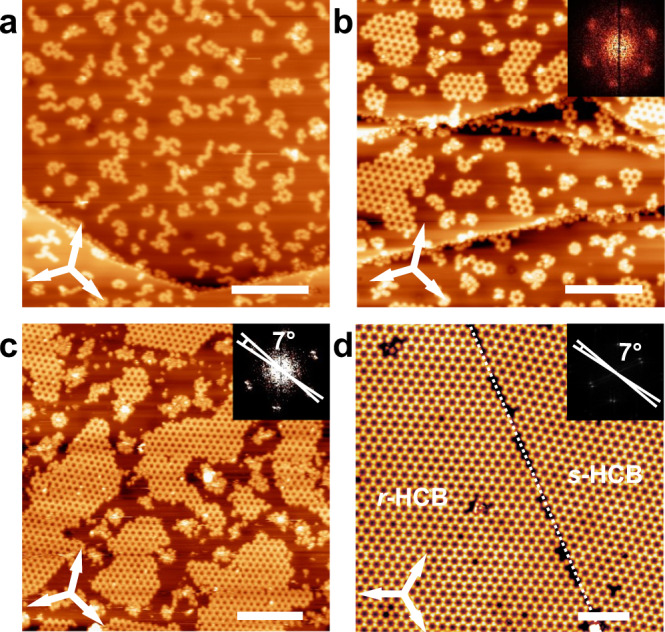
Selective formation of the 2D homochiral HCBs on Ag(111). **a**–**d** STM images reveal the nucleation and the growth of the 2D homochiral HCBs of TBTA on Ag(111). Molecular coverage: **a** 0.2 ML, **b** 0.4 ML, **c** 0.8 ML, **d** 1.0 ML. Tunneling conditions: **a**, *V*_bias_ = 0.6 V, *I*_t_ = 50 pA. **b**, *V*_bias_ = 0.5 V, *I*_t_ = 50 pA. **c**, *V*_bias_ = 2.3 V, *I*_t_ = 60 pA. **d**, *V*_bias_ = 1.0 V, *I*_t_ = 50 pA. Scale bars: **a**–**c** 20 nm. **d** 10 nm.

Furthermore, we investigated the 2D crystallization of TBTA on Ag(111) surface. The symmetry of Ag(111) perfectly matches the homochiral dimeric building motif of the 2D homochiral HCBs and we expected that the lattice symmetry can further modulate the preference of the Br⋯Br contacts based on enantiomeric recognition. The preparation and characterization procedures of the TBTA/Ag(111) samples are the same as the TBTA/Ag(100). Figure 8 displays a series of STM images of TBTA/Ag(111) with different coverage. Small patches of homochiral molecular rings emerge on the surface at low coverage. With a gradual increase in the coverage, the molecular ring structures grow in size and finally completely cover the surface, giving birth to extended 2D homochiral HCBs. The orientation of the molecular rings keeps constant during the 2D crystallization. Except for the homochiral molecular rings and the 2D homochiral HCBs, no other regular structures can be observed on Ag(111) regardless of the molecular coverage. The results are in line with expectations. The Ag(111) substrate perfectly matches with the homochiral dimers and the 2D homochiral HCBs of TBTA. Therefore, TBTA molecules adopt homochiral intermolecular recognition selectively and give birth to exclusive 2D homochiral HCBs on Ag(111). The effect of the substrate on the chiral expression has also been observed in the 2D crystallization of heptahelicene on Cu(111) and Cu(100)^2,43^, which was explained by a different adsorption-sites grid defined by the symmetry as well as the available adsorption sites of the substrates.

In conclusion, we provide a molecular-level understanding of Br⋯Br contacts in molecular recognition and crystallization of a prochiral molecule TBTA on Ag(100) and Ag(111) using STM combined with DFT calculations. We found that the type I Br⋯Br contacts already occur at very low molecular coverage and mediate the homochiral recognition of the molecules on Ag(100). Furthermore, they prevail on the surface during the growth stage and lead to the formation of porous 2D homochiral HCBs. On the contrary, although the type II Br⋯Br contacts also occur at the nucleation stage on Ag(100), they play a limited role in the growth stage and prevail only in the 2D racemic ZZRs produced by the phase transition at high coverage. It is suggested that the match of the homochiral dimers and the 2D homochiral HCBs to the substrate lattice is responsible for the occurrence of type I Br⋯Br contacts at the nucleation and growth stages. By employing Ag(111) that perfectly matches with the homochiral dimers, the 2D homochiral HCBs are selectively produced on the surface. Our finding provides insights into the halogen-mediated interactions in 2D chiral recognition and crystallization.

## Methods

The Ag(100) and Ag(111) substrates (99.999%, Mateck) were cleaned by cycles of Ar^+^ sputtering and annealing at ~650 K in an ultrahigh vacuum (UHV) chamber with a base pressure of about 3 × 10^−10^ mbar. TBTA was purchased from ET Co., Ltd Company. The molecule was thoroughly degassed before being deposited on the surface. Then the molecules were sublimated at ~410 K on a cold Ag(100) or Ag(111) surface (150–200 K). All STM measurements were performed using a scanning tunneling microscope (Unisoku co.) with a tungsten tip at 4.3 K. The STM images were analyzed using WSxM^44^.

The theoretical simulation was performed using first-principles methods, in which the exchange-correlation interaction was approximated by Perdew–Burke–Ernzerhof (PBE)^45^ parameterized functional and electron-ion interaction was treated by the projector augmented wave (PAW) potential^46,47^. Dispersion interactions were considered by Grimme’s D3 correction^48^. The cutoff energy for the plane-wave expansion was set to 450 eV. For the TBTA molecules, a single *k*-point was used. In all structures, the energy and force on each atom were relaxed smaller than 1.0 × 10^−6^ eV and 0.01 eV/Å, respectively. STM images were simulated using the Tersoff-Hamann approximation^49^ with the implementation by ref. 50.

### Reporting summary

Further information on research design is available in the Nature Research Reporting Summary linked to this article.

## Supplementary information

Supplementary Information

Reporting Summary

## Data Availability

The data that support the findings of this study are available from the corresponding authors upon request.
