# Supplementary material for: Evolution of Br⋯Br contacts in enantioselective molecular recognition during chiral 2D crystallization
Source: Nat Commun. 2022 Oct 4;13:5850. doi: 10.1038/s41467-022-33446-y (PMC9532412; doi:10.1038/s41467-022-33446-y)
Supplement: Supplementary file 1 — Supplementary Information [file 41467_2022_33446_MOESM1_ESM.pdf]

Supplementary Information

Evolution of Br···Br contacts in enantioselective molecular recognition during chiral 2D crystallization

Zhen-Yu Yi,[1,2] Xue-Qing Yang,[1,3] Jun-Jie Duan,[1,2] Xiong Zhou,[4] Ting Chen,[1,*] Dong Wang,[1,2*] Li-Jun Wan[1,2*]

[1] CAS Key Laboratory of Molecular Nanostructure and Nanotechnology, CAS Research/Education Center for Excellence in Molecular Sciences, Beijing National Laboratory for Molecular Sciences (BNLMS), Institute of Chemistry, Chinese Academy of Sciences, Beijing 100190, China

[2] University of Chinese Academy of Sciences, Beijing 100049, China

[3] College of Chemistry and Chemical Engineering, Hubei University, Wuhan, 430062, China

[4] Beijing National Laboratory for Molecular Sciences (BNLMS), College of Chemistry and Molecular Engineering, Peking University, Beijing 100871, China

*e-mail: chenting@iccas.ac.cn, wangd@iccas.ac.cn, wanlijun@iccas.ac.cn

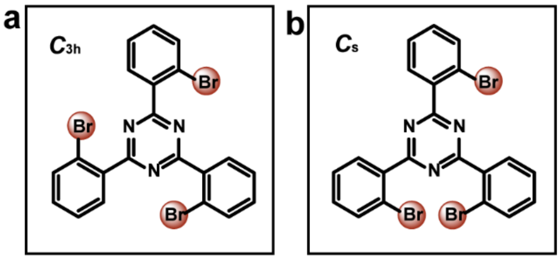

Supplementary Fig.1 Possible conformations of TBTA. **a** The C_{3h}-TBTA conformation. **b** The C_s-TBTA conformation.

According to the orientation of the Br atoms, there are two possible conformations for TBTA, i.e., the C_{3h}-TBTA and the C_s-TBTA. DFT simulations suggest the energy of C_s-TBTA is 0.11 eV higher than that of C_{3h}-TBTA, which may be due to the steric hindrance between the Br atoms.

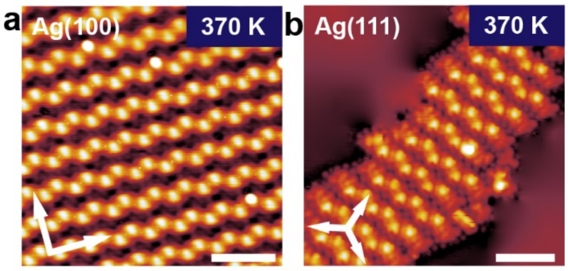

Supplementary Fig. 2 Formation of the organometallic intermediates of TBTA after annealing treatments. **a** STM image of TBTA/Ag(100) after annealing at 370 K. **b** STM image of TBTA/Ag(111) after annealing at 370 K. Tunneling conditions: **a** V_{bias} = 0.11 V, I_t = 50 pA. **b** V_{bias} = 0.19 V, I_t = 100 pA. Scale bars: **a, b** 4 nm.

To verify that TBTA remains intact under the conditions of our measurements, we conducted stepwise annealing of TBTA both on Ag(100) and Ag(111) to monitored its chemical transformation. The results demonstrate that no new structure appears on the surface after annealing at room temperature for 15 mins. After annealing at 370 K, 1D chains with alternating bright and dark dots appear, which are attributed to the oligomers of organometallic intermediates (Supplementary Fig. 2a). Stepwise annealing of the TBTA/Ag(111) reveals similar results. Specifically, no new structure appears on the surface after annealing the sample at room temperature. After annealing treatment at 370 K, 1D chains of the organometallic intermediates are formed, which are surround with bright dots corresponding to the Br atoms (Supplementary Fig. 2b). The

results indicate that the dehalogenation of TBTA and the formation of organometallic intermediates does not occur up to room temperature both on Ag(100) and Ag(111).

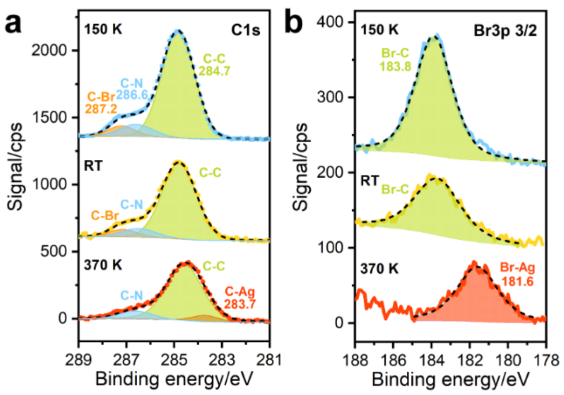

Supplementary Fig. 3 XPS spectra of TBTA on Ag(100). a C 1s spectra of TBTA/Ag(100) at 150 K, room temperature, and 370 K. **b** Br 3p spectra of TBTA/Ag(100) at 150 K, room temperature, and 370 K.

To elucidate the chemical states of TBTA on Ag(100) at different temperatures, we performed XPS characterizations. As shown in Supplementary Fig. 3, three peaks located at 284.7 eV, 286.6 eV, and 287.2 eV can be resolved in the C *1s* spectrum of the sample prepared at 150 K, corresponding to the carbon atoms in C-C, C-N, and C-Br bonds, respectively. Only a peak appears at 183.8 eV in the spectrum of Br *3p*, corresponding to the C-Br bond. The XPS signals of C *1s* and Br *3p* spectra do not show obvious change after annealing the sample at room temperature. The results indicate that the TBTA molecule is intact at 150 K and room temperature. With the increase of the annealing temperature to 370 K, the peaks corresponding to the C-Br bond (both in C *1s* and Br *3p* spectra) decrease, and the peaks corresponding to the C-Ag bond (283.7 eV) and the Br-Ag bond (181.6 eV) appear simultaneously. The results consist well with the results revealed by STM measurements, which support that the integrity of TBTA and the absence of debromination in the present study.

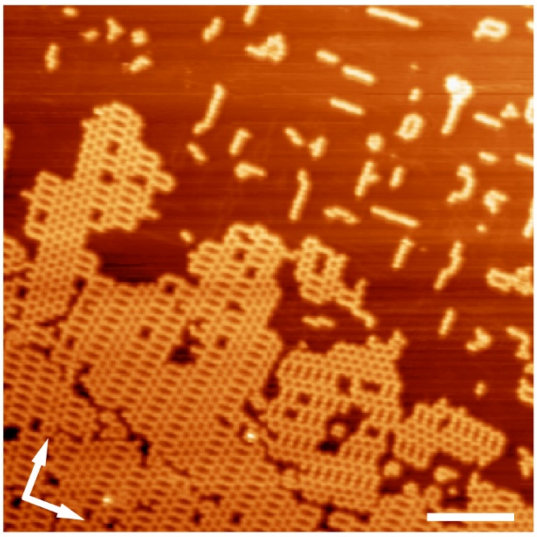

Supplementary Fig. 4 STM image of TBTA/Ag(100) with coverage of 0.4 ML. Tunneling conditions: V_{bias} = 1.0 V, I_t = 50 pA. Scale bars: 20 nm.

The STM image reveal the coexistence of the porous islands grown from the initially formed molecular rings as well as short molecular chains. It can be seen that the length of the molecular chains is comparable to that observed at low-coverage stage, suggesting they do not grow with the molecular coverage.

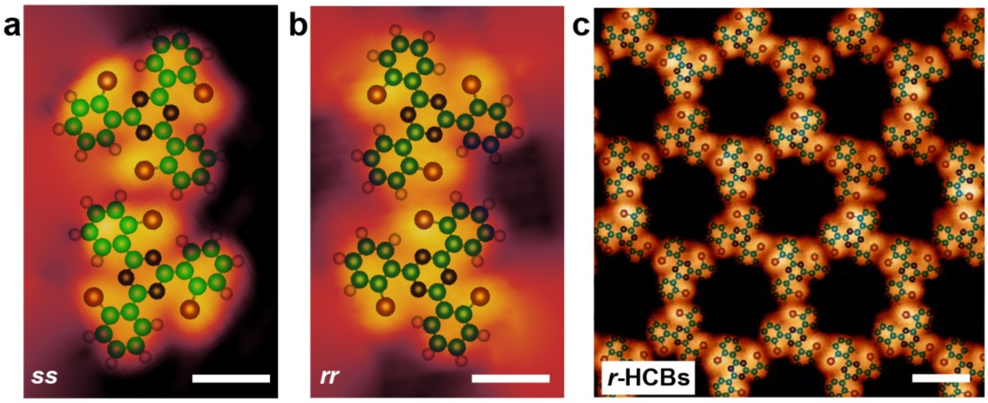

Supplementary Fig. 5 The comparison of the STM images of the homochiral dimers and the HCBs with their molecular models. a *ss*-dimer. **b** *rr*-dimer. **c** *r*-HCBs. Scale bars: **a, b** 0.5 nm**. c** 1 nm.

To compare the STM data and the molecular models of the homochiral dimers and the HCBs, we superimposed the molecular models upon the high-resolution STM images, as shown in Supplementary Fig. 5. One thing to note is that the Br atoms appear as very bright spots in STM images; the phenyl moieties, by contrast, appear as faint spots. Therefore, we judge the match between the molecular models and the STM

images mainly based on the positions of the Br atoms and the central triazine rings. It can be seen that the molecular models of the homochiral dimers and the *r*-HCBs consist well with their molecular models.

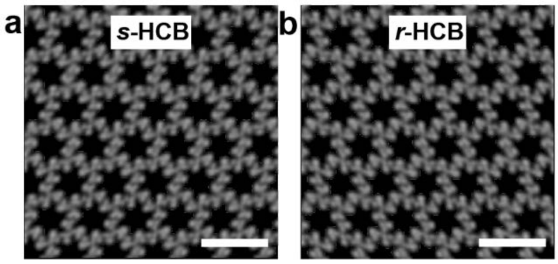

Supplementary Fig. 6 The simulated STM images of the 2D conglomerates. a *s*-HCBs. **b** *r*-HCBs. Scale bars: **a, b** 4 nm.

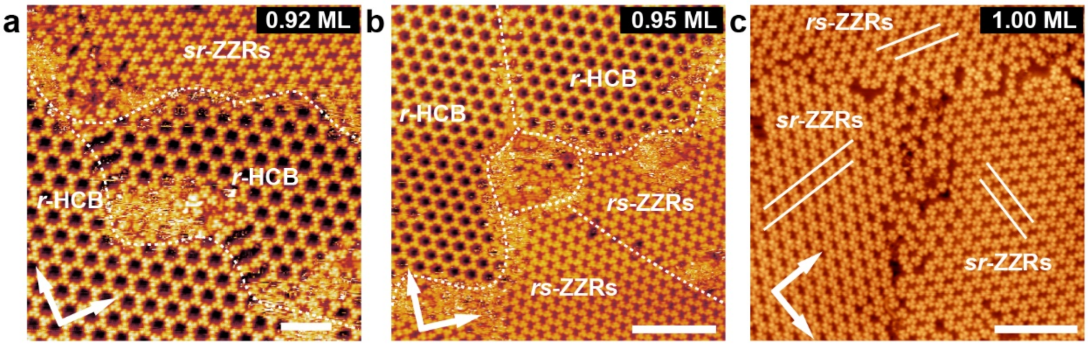

Supplementary Fig. 7 Transition from the 2D homochiral HCBs to the 2D racemic ZZRS. a-c STM images of TBTA/Ag(100) with coverage of 0.92 ML, 0.95 ML, and 1.00 ML, respectively. Tunneling conditions: **a** V_{bias} = -1.6 V, I_t = 100 pA. **b** V_{bias} = 0.5 V, I_t = 50 pA. **c** V_{bias} = -0.3 V, I_t = 50 pA. Scale bars: **a, b, c** 10 nm.

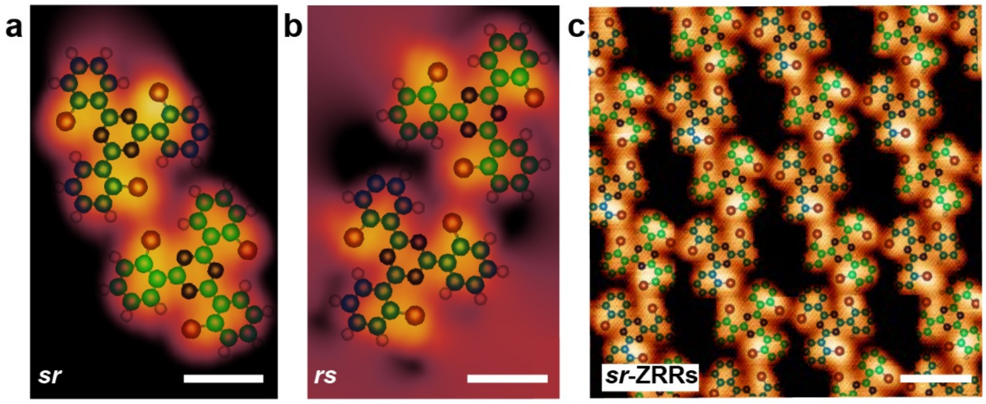

Supplementary Fig. 8 The comparison of the STM images of the heterochiral dimers and the ZZRs with their molecular models. a *sr*-dimer. **b** *rs*-dimer. **c** *sr*-ZZRs. Scale bars: **a, b,** 0.5 nm**. c** 1 nm.

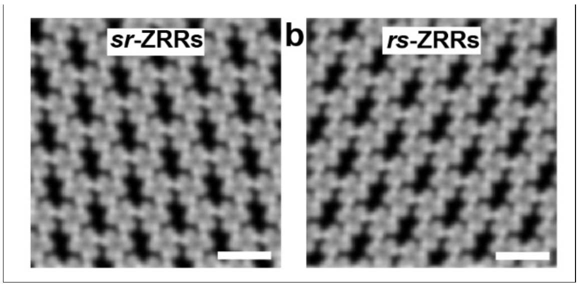

Supplementary Fig. 9 The simulated STM images of the 2D racemates. a *sr*-ZRRs. **b** *rs*-ZZRs. Scale bars: **a, b** 2 nm.

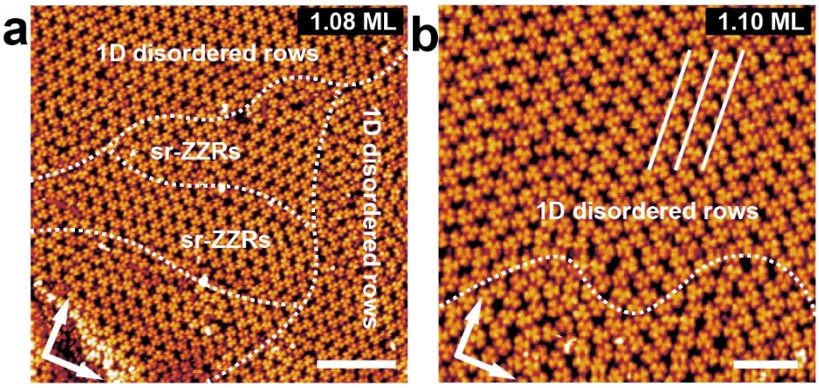

Supplementary Fig. 10 Transition from the 2D racemic ZZRs to the 1D-disordered racemic structures. a-b STM images of TBTA/Ag(100) with coverage of 1.08 ML and 1.10 ML, respectively. Tunneling conditions: **a** V_{bias} = -0.09 V, I_{t} = 150 pA. **b** V_{bias} = -1 V, I_{t} = 100 pA. Scale bars: **a** 10 nm. **b** 4 nm.

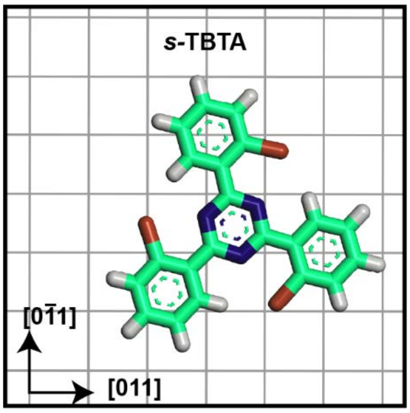

Supplementary Fig. 11 The optimized adsorption configurations of *s*-TBTA on Ag(100).

The orientation of the 2D homochiral HCBs with respect to the substrate lattice keeps constant during the expansion of the HCBs into two-dimensional structure, implying that the molecular orientation in the HCBs is the preferred adsorption orientation. According to the experimental results, it is deduced that the rotation axis of the *s*-TBTA molecule deviates from the <$0\bar{1}1$> direction of the substrate by -13°. We referred to this molecular orientation within the HCBs in building the initial molecular model for the DFT simulations.

On this basis, we calculated the energy of the TBTA/Ag(100) systems where the centre of the triazine ring in TBTA is located at the hole, the bridge, and the top position of Ag(100). The results indicate that the energy of the *s*-TBTA/Ag(100) system is lowest when the center of the triazine ring is at the hole position of Ag(100), suggesting this adsorption site is favored over the other sites.

After that, we fixed the adsorption site of the center triazine ring at the hole position and calculated the energies of the *s*-TBTA/Ag(100) systems where the molecule shows different orientation with respect to the substrate. The results suggest that *s*-TBTA/Ag(100) in which one of the bromophenyl groups exhibits an offset of -13° with respect to the <$0\bar{1}1$> direction of Ag(100) is energetically favored, which agrees well with the experimental STM results.

Therefore, it is inferred that *s*-TBTA/Ag(100) with the triazine ring at the hole position and one of the bromophenyl groups deviated from the <$0\bar{1}1$> direction of Ag(100) by -13° is the optimized adsorption configuration.

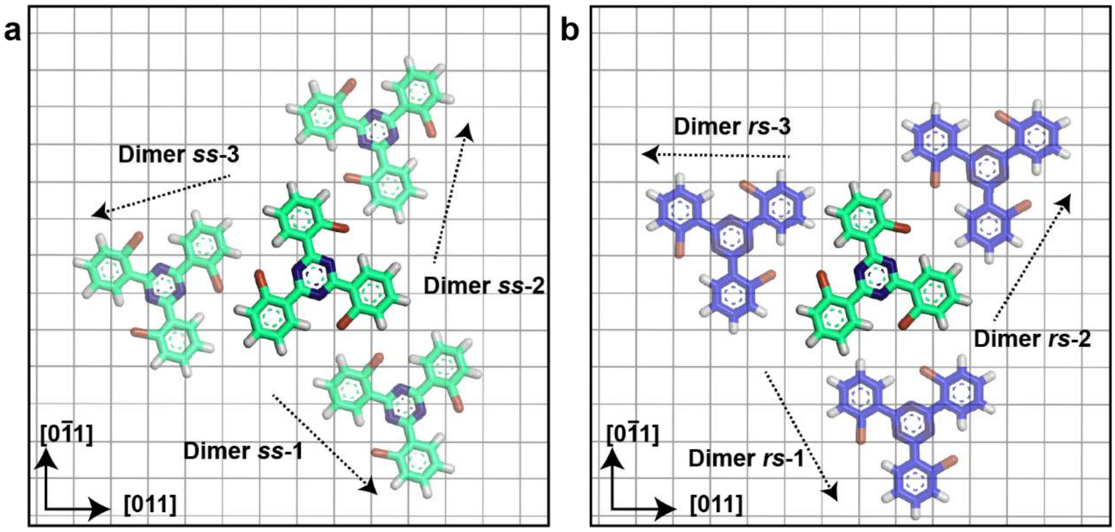

Supplementary Fig. 12 The adsorption configurations of TBTA dimers on Ag(100). a Homochiral *ss*-dimers. **b** Heterochiral *rs*-dimers. The *s*-TBTA located in the center adopts the optimized adsorption configuration. The translucent molecules indicate the possible positions of the other adsorbed molecule to form dimers.

We assumed that the *s*-TBTA adopts the optimized adsorption configuration and explored the effect of the adsorption configuration (adsorption site and orientation relative to *s*-TBTA) of the other molecule on the energy of the *ss*-dimer/Ag(100) or the *sr*-dimer/Ag(100) entity. Considering the orientation with respective to the substrate, three homochiral dimers and three heterochiral dimers could be obtained, named *ss*-1, *ss*-2, *ss*-3, and *rs*-1, *rs*-2, *rs*-3 in Supplementary Fig. 12.

Supplementary Table 1. The energies of the optimized adsorption configuration of TBTA dimers on Ag(100). The energies are relative to the *ss*-1.

**Entry**	***ss*-1**	***ss*-2**	***ss*-3**	***rs*-1**	***rs*-2**	***rs*-3**
**Energy (eV)**	0.00	-0.02	0.06	0.12	0.14	0.08

ss-1, ***ss***-2, ***ss***-3, and ***rs***-1, ***rs***-2, ***rs***-3 represent three homochiral dimers and three heterochiral dimers in Supplementary Fig.12

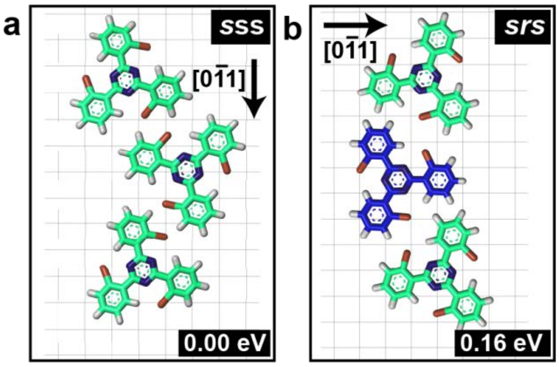

Supplementary Fig. 13 The optimized adsorption configuration of the trimeric clusters of TBTA on Ag(100). **a** Homochiral *sss*-trimer. **b** Racemic *srs*-trimer. The energy is relative to *sss*-trimer/Ag(100).

The molecular models of the trimeric clusters used for DFT calculations were built based on the optimized stable molecular models of *ss*-1 dimer/Ag(100) and *rs*-3 dimer/Ag(100) shown in Supplementary Fig. 12. That is, another *s*-TBTA molecule was added to the optimized *ss*-1 dimer/Ag(100) and *rs*-3 dimer/Ag(100) respectively to be used as the initial molecular models of the *sss*-trimer/Ag(100) and the *srs*-trimer/Ag(100).
